# Heat Emitting Damage in Skin: A Thermal Pathway for Mechanical Algesia

**DOI:** 10.3389/fnins.2021.780623

**Published:** 2021-10-28

**Authors:** Tom Vincent-Dospital, Renaud Toussaint, Knut Jørgen Måløy

**Affiliations:** ^1^SFF Porelab, The Njord Centre, Department of Physics, University of Oslo, Oslo, Norway; ^2^Université de Strasbourg, CNRS, Institut Terre & Environnement de Strasbourg, UMR 7063, Strasbourg, France

**Keywords:** rupture, pain, transient receptor channels, heat dissipation, skin

## Abstract

Mechanical pain (or mechanical algesia) can both be a vital mechanism warning us for dangers or an undesired medical symptom important to mitigate. Thus, a comprehensive understanding of the different mechanisms responsible for this type of pain is paramount. In this work, we study the tearing of porcine skin in front of an infrared camera, and show that mechanical injuries in biological tissues can generate enough heat to stimulate the neural network. In particular, we report local temperature elevations of up to 24°C around fast cutaneous ruptures, which shall exceed the threshold of the neural nociceptors usually involved in thermal pain. Slower fractures exhibit lower temperature elevations, and we characterise such dependency to the damaging rate. Overall, we bring experimental evidence of a novel—thermal—pathway for direct mechanical algesia. In addition, the implications of this pathway are discussed for mechanical hyperalgesia, in which a role of the cutaneous thermal sensors has priorly been suspected. We also show that thermal dissipation shall actually account for a significant portion of the total skin's fracture energy, making temperature monitoring an efficient way to detect biological damages.

## Introduction

The toughness of matter is often characterised by its energy release rate (Griffith, 1921), that is, by the amount of mechanical energy which is dissipated by the rupture of a surface unit of a given solid matrix. Among other dissipation mechanisms (e.g., Rice and Drucker, 1967; Morrissey and Rice, 1998), running cracks tend to emit some heat, a phenomenon which has long been reported and studied by the fracture physics community (e.g., Irwin, 1957; Rice and Levy, 1969; Fuller et al., 1975; Pallares et al., 2012). In various materials, such as PMMA (polymethyl methacrylate) and acrylic adhesives (Vincent-Dospital et al., 2020b), or such as paper (Toussaint et al., 2016), this heat actually accounts for a significant portion (i.e., from 10 to almost 100%) of the total energy release rate, that is, it accounts for a significant portion of these materials' strength. More than a simple energetic loss, the heat dissipation have been suspected to lead to potentially paramount secondary effects, which are still actively debated. These effects notably include the brittleness of matter (Marshall et al., 1974; Carbone and Persson, 2005; Braeck and Podladchikov, 2007; Toussaint et al., 2016; Vincent-Dospital et al., 2020a) or the instability of some seismic faults (e.g., Wibberley and Shimamoto, 2005; Rice, 2006; Sulem and Famin, 2009) through various thermal weakening phenomena.

Recently, we have also suggested a theory (Vincent-Dospital and Toussaint, 2021) that the damage-induced heat in biological tissues could be responsible for a degree of mechanical pain in the human body. Indeed, should the heat dissipation be large enough, it may be detected by the thermo-sensitive nociceptors of sensory neurons, and, in particular, by some of the so-called TRP proteins. Action potentials (i.e., electrochemical signals) may thus be triggered in the nervous system. TRPs (standing for Transient Receptor Potential cation channels—e.g., Wang and Woolf, 2005) are proteins expressed in many cells of the cutaneous tissue (Tóth et al., 2014), and, in particular, at the surface of sensory neurons. Their ability to gate ions through cells' membranes (Nilius et al., 2005) is temperature dependent, and different TRP types react to different cold or warm temperature ranges. As we are here interested in the detection of hot anomalies around running fractures, let us now focus on the TRPs that have been reported to be heat sensitive in skin. In our previous theoretical work (Vincent-Dospital and Toussaint, 2021), the role of TRPV3 and TRPV1 was considered. The former (TRPV3) is sensitive to temperatures in the normal biological range (i.e., 30–40°C), making it, likely, responsible in part for the feeling of warmth (Xu et al., 2002). The latter (TRPV1) starts to activate at more painful temperatures above about 43°C (e.g., Caterina et al., 2000). Additionally to TRPV3, other nociceptors, TRPV4 and TRPM2, are also responsive to subtle temperature changes in the normal physiological range (Güler et al., 2002; Kashio and Tominaga, 2017), and, in addition to TRPV1, TRPM3 has also been evidenced to detect higher painful temperatures (Vriens et al., 2011). Completing the ranges of these various heat sensors, TRPV2, activates at the most noxious temperatures above 52°C, although it has been suggested that this particular sensor has little role in mechanical or thermal pain (Park et al., 2011). Generally, the role of TRPs is commonly admitted in thermal sensing, and some also react to specific chemicals, for instance contained in “hot” pepper or “cool” mint (e.g., Wang and Woolf, 2005). The role of TRPs in the feeling of mechanical pain, which is here of main interest, has also been previously suspected. Thermal and mechanical pains were shown to be coupled in human subjects (Culp et al., 1989), with a threshold to feel mechanical pain that decreases at a higher ambient temperature. Incidentally, cooling is sometimes used for the anesthesia of cutaneous and non-cutaneous tissues prior to medical mechanical injections (e.g., Smith, 2010; Besirli et al., 2020). In rodents, the drug-induced inhibition and activation of TRPV1 and TRPV3 has also proven to, respectively, reduce or increase mechanical hyperalgesia (Culp et al., 1989; Pomonis et al., 2003; Walker et al., 2003; McGaraughty et al., 2017), that is, the decreased threshold to feel mechanical pain after a first stimulus. It should however be noted that the neuroscience community is not unanimous when it comes to the involvement of TRPV1 in mechanical hyperalgesia (e.g. Urano et al., 2012). Finally, the involvement of mammalian TRPV4 in the direct mechanosensation of living organisms has also been shown (Liedtke et al., 2003).

In this work, we experimentally show that the rupture of skin can generate heat anomalies that are in the sensing ranges of the mentioned TRP proteins, on time and space scales that are similar to those of these nociceptors sensibility (Oaklander, 2001; Yao et al., 2010; Liu and Qin, 2019). We thus confirm the relevance of the proposed new thermal pathway for mechanical algesia. We tear pork skin samples, assumed to be a reasonably good model for human skin (e.g., Debeer et al., 2013; Thomsen et al., 2014; Ranamukhaarachchi et al., 2016), in front of an infrared camera and report temperature elevations, over hundreds of milliseconds, of a few degrees to tens of degrees depending on the skin samples and the damaging rate. With a normal skin temperature of about 35°C (e.g., Saxena and Arya, 1981; Otsuka et al., 2002), such thermal anomalies shall indeed open the TRP channels, and we here discuss both direct algesia and hyperalgesia scenario. We characterise the relationship between damage velocity and local temperature elevation, suggesting that a minimal fracture velocity of about 1 cm s^−1^ may be needed for strong thermo-mechanical pain to actually be at play. We also provide the energy release rate of our samples, ~135 kJ m^−2^ in average, and, with two different methods, we give a coarse estimation of the portion this energy release rate (~3–50%) which actually transforms into heat, when fractures progress in skin. We thus show that heat dissipation is responsible for a non negligible part of the cutaneous strength, actually making temperature monitoring an efficient way to detect mechanical damages in biological tissues.

## 1. Methods

### 1.1. Experimental Set-Up

**Figure 1:**
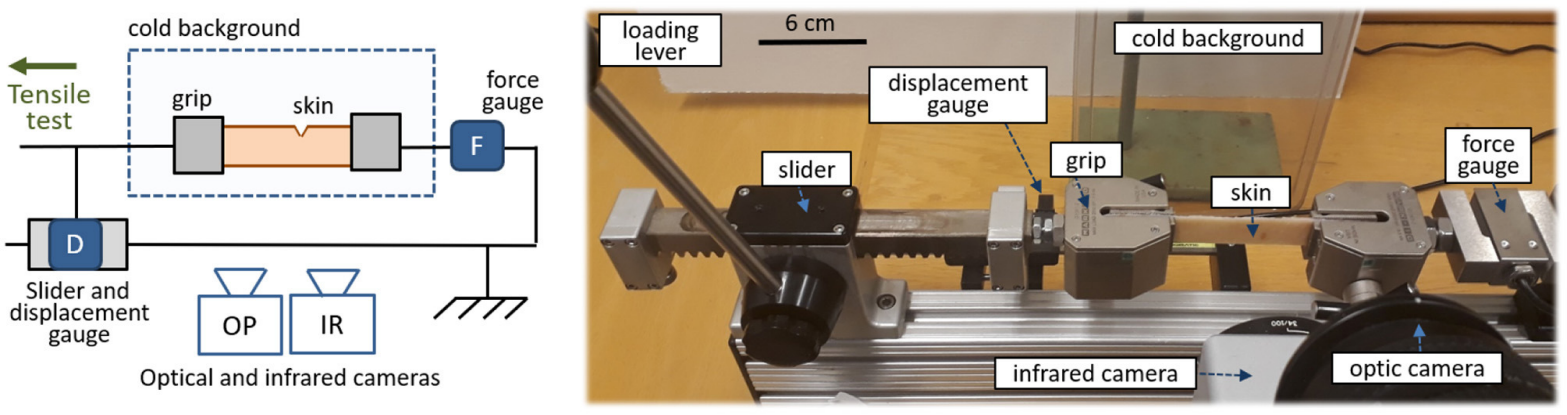
Schematic **(left)** and picture **(right)** of the experimental set-up. Porcine skin is teared in front of an optical and an infrared camera, while both the applied force and displacement are measured. In the real set-up (e.g., by contrast to what is suggested by the schematic) the optical camera is placed above the infrared one.

Let us start by describing the experimental set-up. Most of it is a standard mechanical test bench, a schematic and a picture of which are shown in Figure 1. Porcine skin samples are placed in this horizontal test bench, where they are held at their extremities by two self-tightening wedge grips. This type of grips holds stronger and stronger as the sample they clamp is brought into tension. Mechanical tensile tests are performed on the skin up to its full rupture. To that extent, one of the wedge grips can be displaced along a uniaxial slider with the help of a manual lever. The other grip is fixed. It is attached to a force sensor (Mark-10® MR01-300) that allows force measurements up to 1 N accuracy. The displacement of the moving grip is monitored with a digital scale (Mitutoyo® 572-311-10) having a 30μm precision. The accuracies of the force and displacement sensors are satisfactory in regard to the typical maximal force (~500 N) and maximal displacement (~2 cm) of our typical experimental realisations. An optical camera also records both the set-up and the deforming skin sample. A mono-channel infrared camera (Flir® SC3000), measuring the radiation intensity in the 800–900 nm bandwidth and equipped with a macroscopic lens, is placed in front of the skin. This infrared camera, which is sensitive to temperature changes of fractions of degree, monitors the sample as it is loaded up to rupture. It is the main measurement of these experiments, and we will further discuss, in section 1.3, how the time and space resolution of this particular camera is adequate to the present study. Finally, behind the skin sample, a cold plastic plate, just out of a freezer, acts as a cold background for the infrared images. This ensures that any measured elevation of temperature (i.e., the signal of interest) does arise from the sample itself, and later simplifies the images analysis. This cold plate is not in direct contact with the skin and is placed — arbitrarily — 20 cm away from it, so it does not affect the sample's temperature by direct heat conduction.

Experimental set-ups, similar to the one we have here described (i.e., set-ups which monitor a fracture with an infrared camera), have regularly measured significant temperature elevations in the rupture of various materials (e.g., Fuller et al., 1975; Pallares et al., 2012; Toussaint et al., 2016). However, such set-ups were never used, to our knowledge, for the heat characterisation of rupturing biological tissues.

### 1.2. Skin Samples and Model Limitations

Porcine skin was here chosen as a model for human skin, as they both display similar structural, thermal, and mechanical characteristics (Henriques and Moritz, 1947; Cohen, 1977; Debeer et al., 2013; Ranamukhaarachchi et al., 2016). Such comparison holds particularly well when comparing human skin to the cutaneous tissues of other mammals. The skin that we tested was acquired at a local butchery and originates from the flank or the upper leg of various pig specimens. It is a standard product sold by this butchery, and, thus, no pig was harmed specifically for the need of our experiments. The studied skin included the epidermis (that is, the surface layer of the skin), the dermis and a thin layer of subcutaneous fat (hypodermis). The total skin thickness varied between 1.6 and 2.7 mm, and was measured on each sample with a caliper.

**Figure 2:**
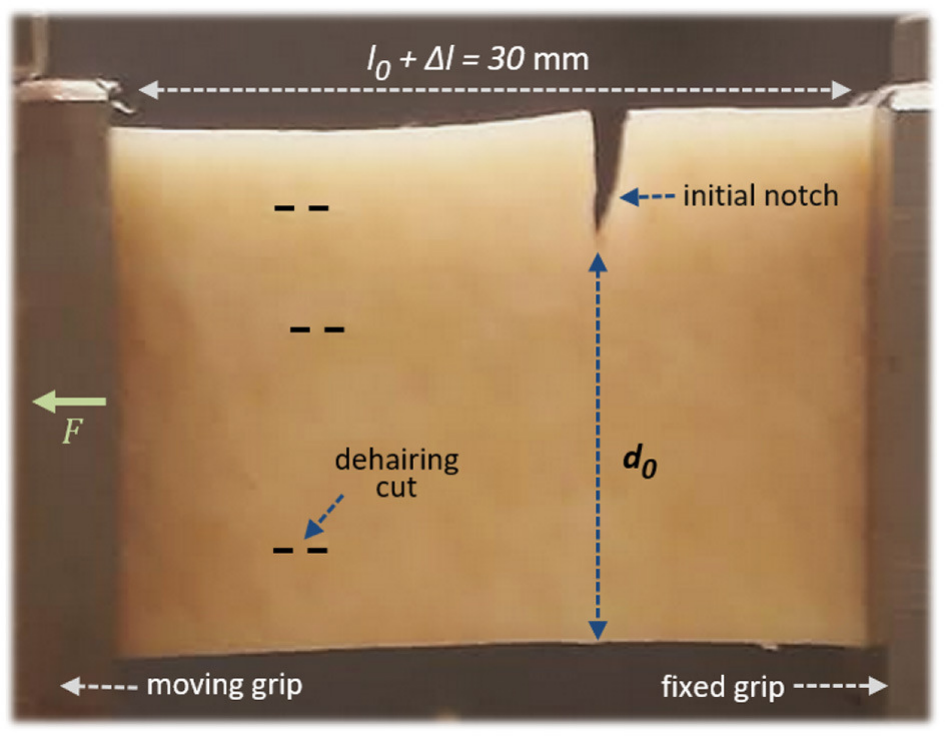
A porcine skin sample installed in the set-up, with the epidermis facing the camera. A little stretch, ~5%, is here applied by the force *F*, so that the nominal sample length between the grips is *l*_0_ = 28.5mm and the stretch is Δ*l* = 1.5mm. The initial unbroken width is denoted *d*_0_. The dashed lines illustrate some barely visible (shallow) cuts on the epidermis, from the dehairing process. The picture's point of view corresponds approximately to that of the infrared camera during the experiments.

The rupture of seven skin specimens (denoted I to VII in this manuscript) was studied, in order to grasp some of the diversity in behaviours of such a biological material. With a scalpel, each skin sample was carved out to be rectangular with width 2.2 cm and length 10–15 cm. The length of the samples simply allowed enough skin to be grabbed by the test bench's wedge grips (grabbing 3.5 cm on each side), to insure the samples stability during the experiments. The force, applied to tear the skin, was applied parallel to this direction. The width of the samples matched both approximately the grips' width and the frame's size of the infrared camera. Figure 2 shows an optical picture of a skin sample installed in the set-up. To ensure that the skin rupture does occur in the frame of the infrared camera, a notch of length 3–6mm was initially cut perpendicularly to the sample's length, to control the initiation of the fracture.

We do not present *in vivo* experiments, for obvious ethical and practical reasons, and we should report that the tested skin went through two initial processes before being acquired that could have slightly alter its mechanical properties. The first of this processes is dehairing (e.g., Scanga, 2005), which sparsely left some cuts on the epidermis (i.e., the shallowest part of our samples). This wear, if significant, should only have weakened the skin, as a perfectly intact skin would be overall tougher and, thus, would likely dissipate more heat upon rupture. In this regard, the results we here present are thus conservative. This being stated, we chose skin samples where the dehairing damage was the less pronounced. The second potentially altering process is freezing, which can cause micro-damages in soft biological tissues (e.g., Leonard et al., 2021). The skin samples were indeed bought frozen and were unfrozen in a ambient temperature water bath during half an hour before running the rupture tests. After this bath, the samples' surfaces were only gently dried with a paper towel. In the case of porcine skin, it was shown (Ranamukhaarachchi et al., 2016) that the fracture energy of the epidermis significantly increases with the freezing/unfreezing process, although by less than an order of magnitude. Conveniently, in the same study (focusing on micro-needle insertions in the epidermis rather than on the full tearing of skin that we here consider) fresh human skin was actually shown to be as tough as unfrozen porcine one (i.e., fresh human skin tend to be slightly stronger than fresh pork skin).

Overall, it should be stated that there is no perfect model for fresh human skin, and we here assume that the various physical parameters, measured on our butchery-acquired samples, are representative of our own cutaneous tissue.

### 1.3. Experimental vs. Biological Resolutions

The displacement's digital measurement was outputted with a 5 Hz rate. The optical camera, recording with a 50 fps (frames per second) rate, hence allowed a faster but less accurate monitoring of the displacement. Depending on the experiment, the force measurement was outputted with a 10–50 Hz rate. Overall, because the total loading time of the samples up to rupture was about 10 s, these rates of measurement were satisfactory for the general characterisation of our mechanical tests. The infrared camera—i.e., the core measurement of our experiments—recorded with a 50 fps frame rate. It had a resolution of 240 × 320 pixels, with a pixel size of 80–90μm depending on the exact camera position and focus of each experimental realisation. This actual pixel size, for given realisations, was calibrated by capturing a hot metallic rod of known diameter, in the same plane as the skin sample on which the camera was priorly focused.

To understand how significant would any heat anomaly be in terms of algesia (pain), it is important to compare the time and space resolutions of our (infrared) thermal measurements to the time and space sensibility of the human nervous system. Based on microscopic observations (e.g., Oaklander, 2001) of the density *D*_*n*_ of neurites in the human epidermis (i.e., the density of the body extensions of neurons), *D*_*n*_~2,000 mm^−2^, one can broadly estimate the nominal distance between two of these neurites to be about $1/\sqrt{D_{n}}\sim$20μm. At the surface of these neurites, the typical response time of some of the TRPs nociceptors to temperature jumps has also been measured, with patch clamp experiments, to range from a few milliseconds to a few tens of milliseconds (Yao et al., 2010; Liu and Qin, 2019). Thus, our experimental resolution in space (~85μm) and time (1/50 Hz = 20ms) should be rather close, yet slightly coarser, to that of neural sensing in the human skin. Any significant temperature change (i.e., as per the TRPs sensitivity) recorded by our set-up should then be able to initiate some action potentials in a live biological system. Oppositely, our infrared camera could miss some of the most accurate details available to the actual neural system on the smallest spatial and temporal scales.

## 2. Results

### 2.1. Temperature Profiles

**Figure 3:**
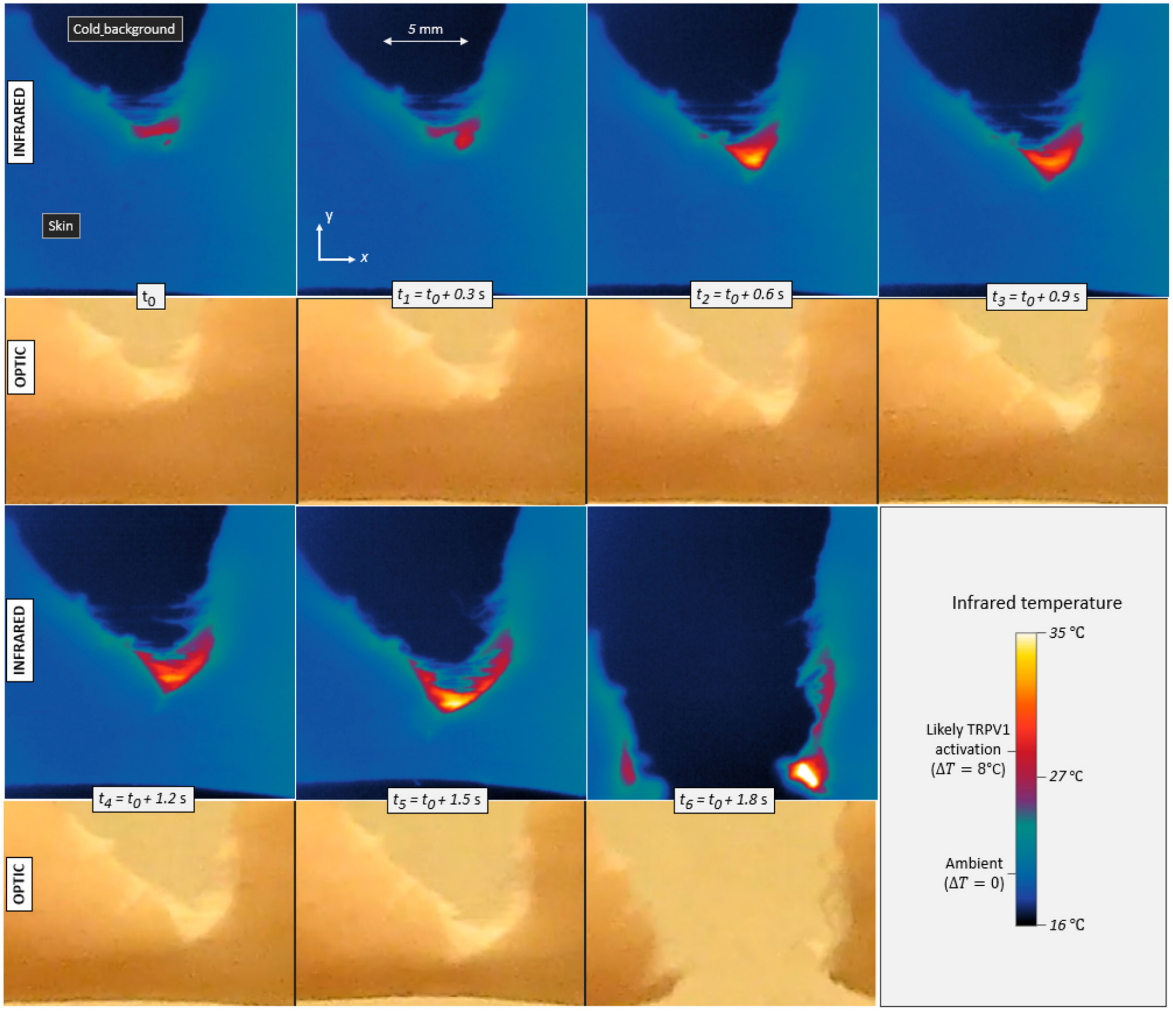
Infrared temperature maps and corresponding optical frames during the propagation of a tensile crack in porcine skin (skin specimen V). Seven different times are displayed with a constant increment in between. The maximum temperature elevation is Δ*T* = 8°C in the first frame (*t*_0_) and up to Δ*T* = 17°C in the last one (*t*_6_), Contrarily to the infrared camera, the optical camera monitored the whole set-up and was not equipped with a macroscopic lens, hence the noticeable difference in image resolution. It also recorded the skin with a slightly different (non-orthogonal) line of sight, as per Figure 1.

Infrared videos, for all experiments, are available to the reader as supplementary materials. Figure 3 shows the maps of measured temperature *T* at seven successive times for skin specimen V. As expected, some heat is emitted by the fracture as it progresses through the cutaneous tissue.

In order to convert the infrared signal to temperatures, skin was assumed to be a black body, that is, to follow Planck's law ( e.g., Jain and Sharma, 1998) and have an emissivity close to 1. In practice, the emissivity of porcine skin may range between 0.94 and 1 (Soerensen et al., 2014). Varying this parameter, it was found that our reported elevations of temperature Δ*T* may hold an error of <10%, and this uncertainty will be irrelevant to our final conclusions.

In Figure 3, one can for instance observe temperature elevations up to 17°C (±1°C). This magnitude is significant with regard to general mammal biology and, more specifically, with regard to the sensitivity of given neuronal thermal sensors. Indeed, assuming a normal inner temperature of about 35°C (i.e., for live subjects), elevations of ~8°C and more should be enough to trigger TRPV1 (and likely TRPM3), and elevations above about 17°C should trigger TRPV2 (e.g., Wang and Woolf, 2005). Additionally, fast thermal elevations of a few degrees Celsius could also excite TRPV3, TRPV4 or TRPM2. It was shown (Xu et al., 2002), in particular, that TRPV3 produces a higher bio-current intensity for faster rises in temperature, and, for temperature elevations of a few degrees, we here measured heating rates of up to 200°C s^−1^. The thermal anomalies typically spread over a few millimetres across and along the crack trajectory, so that many neural receptors (at the surface of about 10^4^ neurites) could likely sense it. As priorly discussed, the typical spacing between two neurites should indeed be in the tens of micrometer range. Appendix A presents some temperature profiles measured in the rupture of the six other skin specimens. Similar orders of magnitude are observed, although a variety of patterns shows in the temperature maps. The maximal temperature elevation Δ*T*_max_, which was recorded during each test, is indicated in Table 1. In all occurrences, it exceeds the TRPV1 threshold and sometimes exceeds that of TRPV2.

**Table 1:** Summary of various physical quantities estimated on each skin specimen.

**Skin** ** specimen**	**h** ** (mm)**	***l*_0_** ** (mm)**	***d*_0_** ** (mm)**	** ${\bar{\sigma }}_{\text{f}}$ ** ** (MPa)**	**E** ** (MPa)**	**Δ*T*_max_** ** (^**°**^C)**	**G** ** (kJ m^**−2**^)**	**ϕ** ** (−) (%)**
I	2.0	65	18	15 ± 2	110 ± 20	24 ± 1.5	130 ± 20	~30
II	2.0	71	16	13 ± 2	31 ± 6	22 ± 1.5	160 ± 20	~5
III	1.8	29	18	10 ± 2	35 ± 6	22 ± 1.5	80 ± 10	~30
IV	1.6	32	17	9 ± 2	48 ± 8	19 ± 1	80 ± 10	~15
V	2.7	25	17	12 ± 2	20 ± 4	17 ± 1	150 ± 20	~30
VI	2.0	32	14	19 ± 3	33 ± 6	12 ± 1	210 ± 30	~15
VII	2.4	42	19	10 ± 2	35 ± 6	16 ± 1	135 ± 20	~50

*${\bar{\sigma }}_{\text{f}}$ is the mean stress at rupture, E is the skin's Young modulus, ΔT_max_ the maximal temperature elevation recorded during a test, G the mean energy release rate and ϕ the mean thermal efficiency in the dissipation of G. As explained in the text, ϕ should not be interpreted beyond its order of magnitude. For reference, the initial samples geometry (i.e., as per Figure 2) is also provided, with accuracy ±0.15 mm for h and ±0.5 mm for l_0_ and d_0_*.

### 2.2. Mean Stress At Rupture and Elastic Modulus

**Figure 4:**
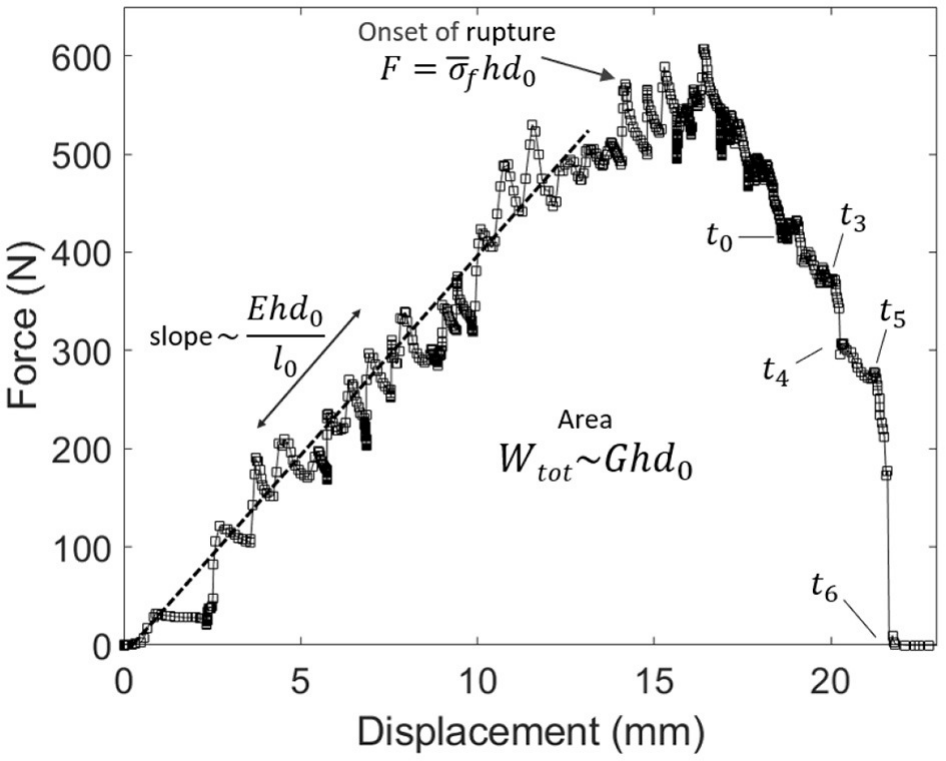
Force vs. displacement plot for the experiment shown in Figure 3 (skin specimen V). Labels *t*_0_–*t*_6_ refer to the times of the frames in this other figure. The relatively linear relation at small displacements allows to invert for the skin's Young modulus *E*. The area below the plot is the total mechanical work *W*_tot_ provided to the sample. The mean stress at the onset of rupture is called ${\bar{\sigma }}_{\text{f}}$.

As we performed relatively standard tensile tests (the most exotic feature being to monitor them with an infrared camera), we here also provide some of the mechanical constants of our skin samples. From Figure 4, showing for specimen V the measured force *F* vs. displacement Δ*l* plot, one can, in particular, estimate a mean stress ${\bar{\sigma }}_{\text{f}}$ at rupture by computing ${\bar{\sigma }}_{\text{f}}=F/\left(hd_{0}\right)$ at the onset of the fracture. Here, *h* is the thickness of the sample and *d*_0_ is its initial unbroken width. Note that this stress value does not account for any stress concentration, so that the actual stress shall reach, locally, higher values, around the initial crack tip or at the scale of the skin's collagen fibers (i.e., the main dry constituent of the dermis). The strength of our samples ranged from about 9 to 19 MPa, which is rather logically comparable to the strength of individual collagen fibers (e.g., Miyazaki and Hayashi, 1999). From the force vs. displacement plots, an elastic (Young) modulus *E* of the skin samples can also be estimated, from the approximately constant ratio *E* = *Fl*_0_/(*hd*_0_Δ*l*) that holds as the sample is loaded elastically. In this expression, *l*_0_ is the initial sample length between the two grips. We derived *E* in the range of 20–110 MPa. For each skin specimen, Appendix A shows the force and displacement plots and Table 1 summarises the samples initial geometry (e.g., *l*_0_, *d*_0_, and *h*), the computed values for the mean stress at rupture ${\bar{\sigma }}_{\text{f}}$ and the cutaneous Young modulus *E*.

Although not the core interest of the present study, it is satisfying that both the values of *E* and ${\bar{\sigma }}_{\text{f}}$ that we report are compatible with other studies of *ex vivo* (e.g., post-mortem) skin samples (e.g., Ní Annaidh et al., 2012). It is however to be noted that significantly lower elastic modulus, in the range of 1Mpa, were also reported for human and porcine skins (e.g., Ranamukhaarachchi et al., 2016). Extensively discussed in the literature (e.g., see Ní Annaidh et al., 2012), the causes for such variability may include the inherent spread in the properties of biological materials, the differences in testing methods (in particular for *ex vivo* vs. *in vivo* samples), or anisotropy in the skin's structure. It may also lie in the dependence of the elastic modulus with the tissue's deformation rate, as skin is a viscoelastic, rather than a purely elastic, material (Rodriguez et al., 2021).

### 2.3. Energy Release Rate

The rise in skin temperature, which we are here mainly interested in, accounts for a portion of the dissipated energy, as the rupture progresses. The total mechanical work that was provided during a tensile test is given by


(1) $$\begin{array}{l} W_{\text{tot}}=\int_{\text{Δ}l=0}^{+\infty }F\text{d}\Delta l, \end{array}$$

that is, the area below the measured force vs. displacement curve (i.e., see Figure 4). By definition, the mean energy release rate of skin *G* can then be derived as


(2) $$\begin{array}{l} G\sim \frac{W_{\text{tot}}}{hd_{0}}, \end{array}$$

where *hd*_0_ is the final created surface upon full sample rupture. The estimated *G* is shown for each skin specimen in Table 1 and is in the 80–210 kJ m^−2^ range (135 kJ m^−2^ in average for all samples, with a significant standard deviation of 35 kJ m^−2^).

A first remark is that the magnitude of *G*, here reported for the tearing of skin, is significantly higher than that reported for the scissors cutting of skin (Pereira et al., 1997), which is about 2kJ m^−2^. It is also higher than the likely energy release rate of individual polymeric fibers (e.g., of collagen fibers, which compose most of the cutaneous tissue), which should be in the order of 1kJ m^−2^ (Porter et al., 2013). Likely, this difference translates that, contrarily to cutting, tearing is a process involving some fibre-to-fibre interactions rather than only processes below the fiber's scale, with the (likely heat-emitting) friction between these fibers known to account for most of the tissue toughness (e.g., Yang et al., 2015).

Another remark is that Vincent-Dospital et al. (2021) proposed the energy release rate of a material to be related to the core length scale *l* at which most of the energy is dissipated:


(3) $$\begin{array}{l} l\sim \frac{a^{3}G}{2u}, \end{array}$$

where *a*~2 Å is the typical size of a molecular link and *u*~1 eV the typical magnitude of its energetic cohesion. Satisfyingly, in our case, this value is in the micrometer range [l ~3μm in average, although Equation (3) only provides an order a magnitude]. Such value is a typical length scale for the diameter of collagen fibres (Verhaegen et al., 2012), which tends to confirm the importance of this scale in the tearing of skin. Note that, because this size is small compared to the extend of our measured thermal anomaly (i.e., Figure 3), it suggests that most of this anomaly is subsequent to the heat diffusion at larger scale, and not directly related to the intrinsic size of the heat sources *l*.

### 2.4. Approximate Thermal Energy Budget

To estimate the portion ϕ of *W*_tot_ that was dissipated as heat, we computed the rise in internal energy *U* that could be captured by the infrared camera as


(4) $$\begin{array}{l} U\left(t\right)=\rho ch\iint_{S}\text{Δ}T\left(x,y,t\right)\text{d}s. \end{array}$$

In this expression, *c* is the heat capacity of porcine skin, ~3.2 kJ K^−1^ kg^−1^ (Henriques and Moritz, 1947; Giering et al., 1996), *S* is the surface of the sample available to the camera, *x* and *y* are the 2D-coordinates of the infrared frames (i.e., see Figure 3), and d*s* = d*x* × d*y* is the elementary surface unit (i.e., the surface of one infrared pixel, in this case). We assessed, with a simple scale, the volumetric mass ρ of samples of various measured volumes to be 1, 150 ± 50 kg m^−3^.

**Figure 5:**
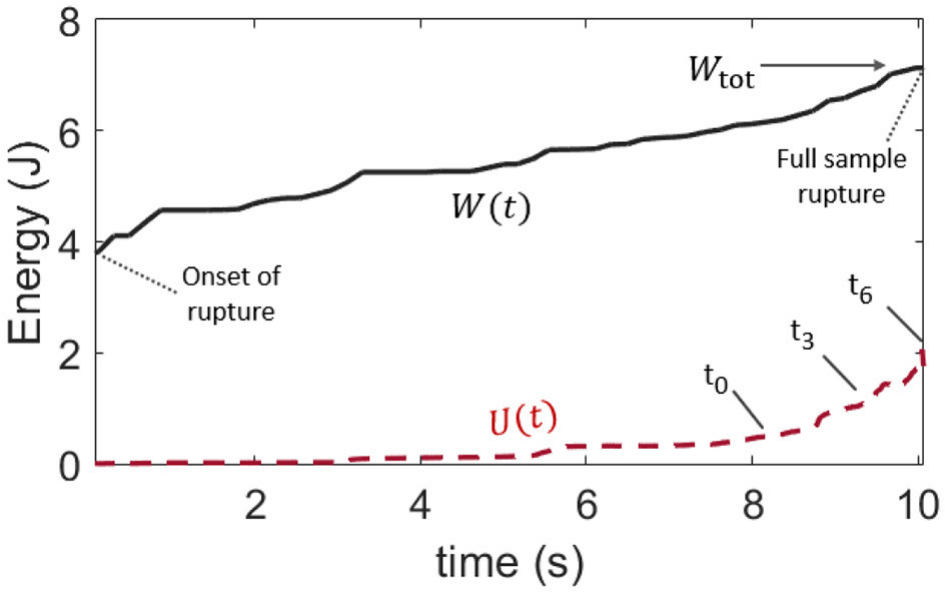
Comparison of the time evolution of the provided mechanical load *W*, as per Figure 4, and that of the rise in internal (thermal) energy *U* in the skin sample, as per Equation (4). This plot is for skin specimen V. Labels *t*_0_ to *t*_6_ refer to the frames of Figure 3. The mean observable thermal efficiency, which is the ratio ϕ = *U*/*W*_tot_ at the end of the rupture, is here about 30%.

The time evolutions of both the mechanical work *W*(*t*) provided to the teared samples (which is defined the same way as *W*_tot_, but for an ongoing rupture) and of the thermal energy *U*(*t*) are shown in Figure 5, for the same experiment (V) whose results are displayed in Figures 3, 4. They are also shown for the other skin specimens in Appendix A. The mean thermal efficiency ϕ, for the complete rupture, can then be computed at the end of the experiment, when all of *W*_tot_ as been dissipated. It is defined as the final *U*/*W*_tot_ ratio and ranges between 5% and 50% depending on the skin sample.

Such a wide range for ϕ does not come as a surprise. In addition to the likely complexity in the rupture of skin, and to the intrinsic diversity in this biological material, the total dissipated energy and the thermal energy were only broadly estimated. Indeed, our definition of *W*_tot_ [i.e., Equation (1)] is not fully intrinsic to the studied material and may depend on the loading geometry and sample size. Note that such dependency with the sample size does not however display in Table 1, where *l*_0_ and *G* do not appear to be particularly correlated. Likely, it translates that size effects are small compared to our samples' variability in strength. There are also several hypothesis presupposed by Equation (4) in the computation of *U*, and not all of them may be conservatively respected. First, it is supposed that all of the thermal energy is available to the infrared camera. In this regard, we made sure to compute *U* only as long as the heat conduction through the skin did not obviously transfer this energy out of the camera frame (as the full length of the stretched sample is not monitored by the camera which is only framed around the crack tip). We also verified that the energy exchange with the air surrounding the sample was slow enough to be neglected over the time of observation. The typical time constant for such an air-skin exchange was indeed measured (see Appendix B) to be about 6 minutes when the fracture of a skin sample typically took a few seconds. Likely, the strongest hypothesis behind Equation (4) is that the temperature profile, that is only measured at the surface of the epidermis, holds on the full sample's thickness *h*. In practice, skin is a layered (heterogeneous) material, and significant temperature differences may hold between the epidermis, dermis, and hypodermis. Additionally, in Figure 3, one can observe likely thin fiber bundles around the progressing crack, indicating that the assumption that *h* is a homogeneous thickness is no doubt limited. Finally, as the rupture progresses, the portions of the skin sample lying behind the crack tip gain some freedom in moving outside of the focal plane of the infrared camera, so that the temperature measurement may there be less accurate.

Overall, ϕ should not be interpreted beyond its order of magnitude. Yet, and despite the listed limitations, we compute, in the next section, the thermal efficiency ϕ with a different method and obtain similar results (in order of magnitude) to what we have here reported.

### 2.5. Temperature Elevation vs. Damage Speed

**Figure 6:**
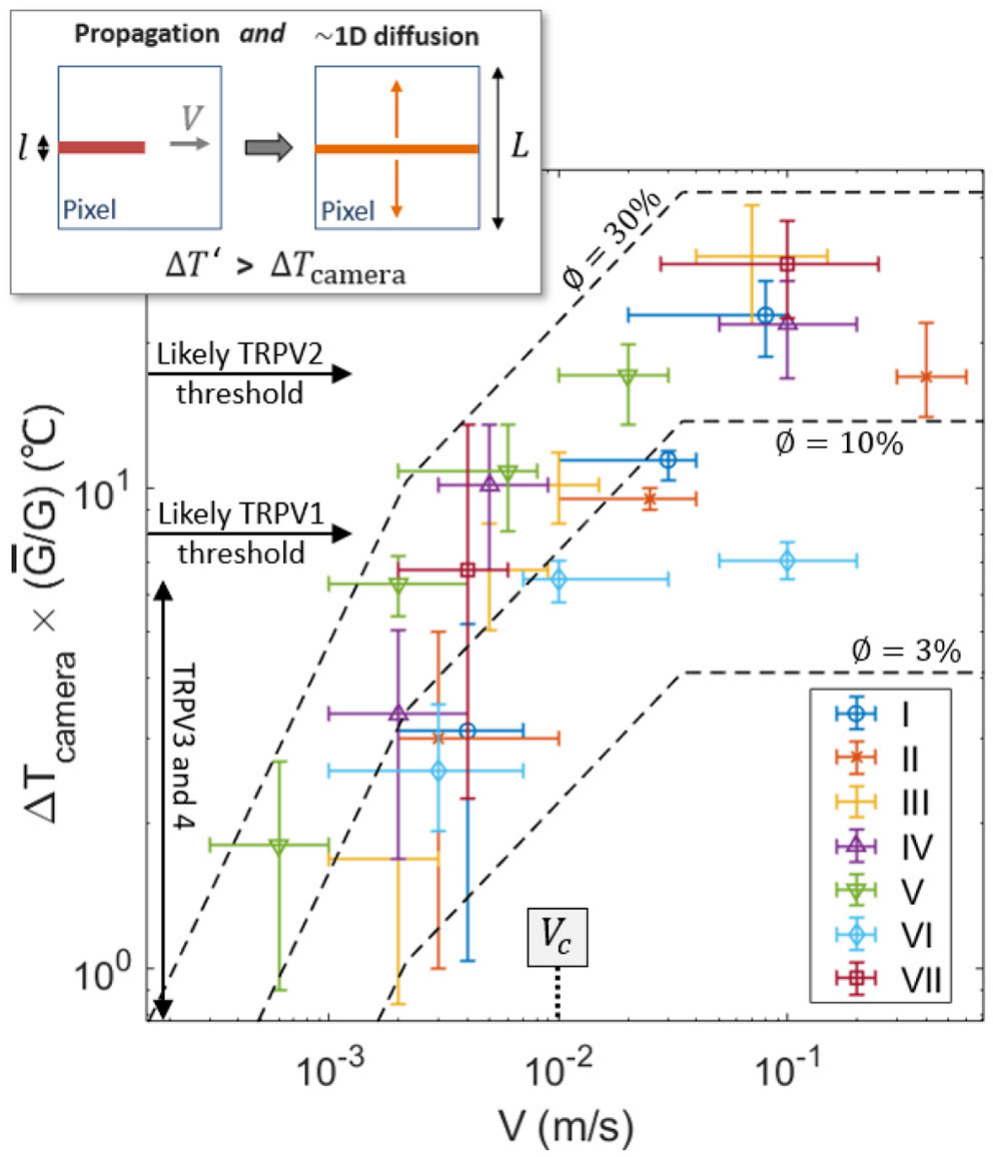
Recorded elevation of temperature Δ*T* at the damage tip as a function of crack velocity (log scales), scaled by $\bar{G}/G$ for each skin sample. Data points for the seven experiments are shown with different symbols (and colors). For reference, the thresholds for the activation of the TRPVs are shown by the plain arrows, assuming an ambient temperature of 35°C for live inner skin. The dashed lines are the model described by Equation (9) using ϕ = 3, 10, and 30%, and with ρ*c* = 3.7 MJ K^−1^ m^−3^, λ = 0.3 J s^−1^ m^−1^K^−1^, $\bar{G}=135$ kJ m^−2^ and *l* = 3μm. The transition velocity *V*_*c*_ predicted by the model [i.e., Equation (8)] is also indicated. The top-left inset illustrates the 1D diffusion hypothesis lying being Equation (9), due to the time scale difference between the crack propagation and the heat diffusion at the camera pixel size.

One can notice, in Figure 3, some correlation between the crack velocity *V* and the magnitude of the temperature anomaly Δ*T*. Compare, for instance, the relative crack advancement and the tip temperature between times *t*_0_ and *t*_1_ and times *t*_5_ and *t*_6_. Figure 6 displays the relationship between the maximal recorded temperature elevation and the crack velocity, as observed during the experiments. To better compare the different experiments, Δ*T* is there rescaled by the ratio $\bar{G}/G$ where $\bar{G}=135$kJ m^−2^ is the mean energy release rate of all the samples. To avoid confusion, we remind here that *G* itself is an average value over the rupture of a unique sample (i.e., derived from Equations 1 and 2). Note that the exact position of the crack tip, which is necessary to define an accurate velocity and which we have manually picked on each infrared frame, is subject to a large incertitude, and the data in Figure 6 thus retains relatively large error bars. A similar trend is yet shown for all skin specimens, with Δ*T* increasing with the fracture velocity.

Such a correlation of temperature elevation with velocity does not come as a surprise, and has been investigated for the rupture of other materials (e.g., Toussaint et al., 2016). Fast cracks tend to be hotter, as less time is then allowed for thermal conduction to efficiently evacuate the excess in heat away from the crack tip (Rice and Levy, 1969; Toussaint et al., 2016; Vincent-Dospital et al., 2020b), where the energy is dissipated. A model based on these considerations has notably shown (see for instance Toussaint et al., 2016) that, at low velocity, the tip temperature elevation [hereafter referred to as $\text{Δ}T_{\text{slow}}^{\prime }\left(V\right)$] should increase linearly with fracture velocity. For faster crack tips, the temperature should however increase slower and slower with *V* [a relation which is hereafter referred to as $\text{Δ}T_{\text{transition}}^{\prime }\left(V\right)$] and eventually reaches a plateau $\text{Δ}T_{\text{plateau}}^{\prime }$ at the highest velocities. The notation Δ*T*′ is here used to differentiate the prediction of this simple model and the actual camera measured temperature elevation Δ*T*. The three asymptotic regimes of the model (Toussaint et al., 2016) are described by:


(5) $$\begin{array}{l} \text{Δ}T_{\text{slow}}^{\prime }\sim \phi G\frac{V}{\lambda }, \end{array}$$

(6) $$\begin{array}{l} \text{Δ}T_{\text{transition}}^{\prime }\sim \phi G\sqrt{\frac{V}{4\pi \rho c\lambda l}}, \end{array}$$

(7) $$\begin{array}{l} \text{Δ}T_{\text{plateau}}^{\prime }\sim \frac{\phi G}{\pi \rho cl}. \end{array}$$

where λ ~ 0.3 J s^−1^ m^−1^K^−1^ is the heat conductivity of skin (e.g., Cohen, 1977) and *l* is the typical length scale over which energy is dissipated and partly transformed into heat.

Assuming that the different physical parameters are relatively independent on velocity, these equations describe a transition between Δ*T*′ ∝ *V*^1^ and Δ*T*′ ∝ *V*^0^, which is highly compatible with the experimental observation (see Figure 6, where Δ*T* increasing by 1.5 orders of magnitude when *V* increases by 3 orders of magnitude). A prediction of the model is that such a transition occurs at velocities around *V* = *V*_*c*_, with


(8) $$\begin{array}{l} V_{\text{c}}\sim \frac{\lambda }{\pi \rho cl}, \end{array}$$

which corresponds to velocities for which the diffusion skin depth $\sim \sqrt{\lambda \left(l/V\right)/\left(\pi \rho c\right)}$ over the intrinsic warming time *l*/*V* is similar to the size *l* of the heat source (Toussaint et al., 2016). Because our experimental data seems to lie in the transition range, Equation (8) is another way of estimating *l*. Indeed, if one uses *V*_*c*_ ~ 1 cm s^−1^, as the central order of magnitude of the velocity of our experimental cracks (i.e., see Figure 6), one obtains *l* in the order of a few micrometers. This value is satisfyingly consistent with the prior estimation from Equation (3) and with the diameter of collagen fibers.

Note that the temperature elevations Δ*T*′, predicted by the model, only hold at the length scale for heat dissipation *l*. That is, they hold at a scale almost two orders of magnitude smaller than the camera pixel size *L*~85 μm. If one then considers that the heat deposited behind the crack tip diffuses perpendicularly to the crack direction up to the pixel size (i.e., assuming 1D diffusion), the temperature elevation available to our infrared camera, when the extra heat has diffused enough, should then be of the order of:


(9) $$\begin{array}{l} \text{Δ}T_{\text{camera}}\left(V\right)\sim \frac{l}{L}\times \{\begin{array}{ll} \text{Δ}T_{\text{slow}}^{\prime }\left(V\right), & \text{if }V\ll V_{c}\\ \text{Δ}T_{\text{transition}}^{\prime }\left(V\right), & \text{if }V\sim V_{c}\\ \text{Δ}T_{\text{plateau}}^{\prime }, & \text{if }V\gg V_{c} \end{array} \end{array}$$

This 1D simplification of heat diffusion is here approximately valid because, for the propagation velocities that we consider, the crack advances by one pixel in a time *L*/*V* ~0.001 to 0.1 s shorter than the typical time *L*^2^ρ*c*/λ ~0.1 s for diffusion at the pixel scale. Therefore, at this scale, the heat transport along the crack direction can be approximately neglected (i.e., see the inset of Figure 6), in particular for the upper part of our measured fracture velocities.

Fitting Equation (9) to the experimental data, as shown in Figure 6, one gets a reasonable match, and, then, a new way of estimating ϕ, that is, a new way of providing an energy budget for the heat dissipation. Indeed, ϕ is the only unknown physical quantity in our model. We found ϕ ~3 to 30%, which is rather compatible with the coarse estimation of section 2.4. Again, one should consider this value as only an order of magnitude, as it is, in practice, highly dependent on a model bound to only be a broad representation of the actual tearing of skin [i.e., the estimation of ϕ is highly dependent on Equations [(5) to (7) and on the re-scaling proposed by Equation (9)]. This model is indeed a continuous mesoscopic approach while skin is highly heterogeneous at the fibre's scale, and simple Fourier conduction (a base for the model) is also known to hold limitations to describe the cutaneous heat transport (e.g., Hooshmand et al., 2015). It nonetheless provides another indication that thermal dissipation accounts for a non negligible part of the strength of the cutaneous tissue.

## 3. Conclusion

In this work, we have demonstrated that the tearing of skin generates temperature anomalies from a few degrees Celsius to tens of degrees Celsius. Unfrozen porcine skin was used as a model for human skin. The recorded heat bursts were observed on space and time scales similar (although slightly coarser) to the expected resolutions of the human neural system (Oaklander, 2001; Yao et al., 2010; Liu and Qin, 2019), and in the sensibility range of the thermal biosensor TRPV3, TRPV4 and TRPM2 (often associated to the feeling of warmth—e.g., Wang and Woolf, 2005), that of TRPV1 and TRPM3 (that have been associated to thermal pain—e.g., Wang and Woolf, 2005; Vriens et al., 2011) and that of TRPV2 (which we chose to here include for completeness, although the role of this protein in thermal sensing is uncertain—e.g., Park et al., 2011). A novel—thermal—pain pathway is thus shown to be likely involved in the reporting of mechanical damages to the nervous system, in particular when the damage rate is fast enough. Indeed, the elevation of skin temperature increases with the damaging rate, and our infrared camera could spot temperature elevations that have the ability to trigger TRPV1 for crack velocities above 1cm s^−1^ and to trigger TRPV2 for even faster cracks (see Figure 6).

In addition to these main results, we have characterised the tearing of our porcine skin samples, by providing their typical mean rupture stress (${\bar{\sigma }}_{\text{f}}\sim$12±4 MPa), their Young modulus (*E*~20–110 MPa depending on the sample), their mean energy release rate (*G*~135±35 kJ m^−2^), the heat energy release rate (from a few percent of *G* to up to 50% of *G*, with a most representative value above 10%), and the typical length scale for the release of heat [*l* in the micrometre range as per Equations (3) and (8)].

We finally showed that a simple physical model (Toussaint et al., 2016), accounting for the heat dissipation and diffusion around cutaneous damages [i.e., Equations (5) to (9)], can quantitatively account for the observed dependency of fracture temperature with fracture speed.

## 4. Discussion

### 4.1. Direct Mechanical Algesia and Secondary Hyperalgesia Mechanisms

From our results, we propose two different thermo-mechanical pain processes. For fastest fractures, direct algesia may arise from the simple activation of TRPV1 (or even TRPV2) at noxious heat level. Slower fractures may not trigger such direct mechanism, although some action potentials may already be send through the nervous system by the warmed-up TRPV3, TRPV4 or TRPM2. It is to be noted that TRPV3 has been shown to present a highly hysteresical sensitivity to thermal anomalies (Liu et al., 2011), responding far better to low-intensity temperature bursts after a first activation at a higher, noxious, level. After a first fast damage, very slow ruptures may thus actively be reported via the stronger activation of TRPV3, in a hyperalgesia process. Although it is debated (e.g. Urano et al., 2012), the mitigation of mechanical hyperalgesia, that is, of the increased sensibility to pain after a first stimulus, when suppressing TRVPs, has actually been one of the indications that first suggested that these proteins should play a role in mechanical pain (Pomonis et al., 2003; Walker et al., 2003; McGaraughty et al., 2017). Similarly to TRPV3, the TRPV2 channel also holds a strong hysteresis (Liu and Qin, 2016) and could also, then, play some role in hyperalgesia, while, oppositely, TRPV1 was shown to provide a consistent response to repeated thermal bursts (Liu and Qin, 2016). Another, not mutually exclusive, mechanism for hyperalgesia, in our framework, could be that inflamed and/or infected tissues around pre-existing wounds tend to exhibit a higher background temperature (by 1–5°C, e.g., Chanmugam et al., 2017), and could then be sensitive to slower fractures, as the TRPV1 threshold (for instance) shall then be easier to reach. It was also suggested that existing injuries facilitate the activation of TRPV1 through the acidification of the skin (Tominaga et al., 1998), as this sensor is also responsive to abnormal pH.

Interestingly, tissue cooling is already used for anesthesia prior to the mechanical injections of treatments (Smith, 2010; Besirli et al., 2020), and a principle lying being such anesthesia methods may lie in preventing any damage-related thermal anomaly to exceed the nociceptors' thresholds. Additionally, we suggest that using highly conductive materials for the design or the coating of needles and other invasive medical tools (i.e., sharps) may help reduce pain, as the heat may then be efficiently transported away from the tissues through the conductive blades. Actually, most sharps are made of stainless or carbon steel, which are already relatively good thermal conductors but less so than some other metals or some other specific materials.

### 4.2. On Possible Higher Temperatures At the Smallest Scales

The pixel size of our infrared measurement (~85μm) was about half an order of magnitude bigger than the typical distance between two neurites (~20μm—e.g., Oaklander, 2001; Vincent-Dospital and Toussaint, 2021). It was also almost two orders of magnitude bigger than the length scale *l*, where the heat is dissipated [here inverted to be in the micrometer range, which is similar to the size of the skin fibers, with two different methods, i.e., with Equations (3) or (8)]. We therefore suggest that, contrarily to what is suggested by Figure 6, cracks propagating at slower velocities than 1cm s^−1^ may already be locally hot enough to trigger direct algesia. Indeed, and although this was not here measured, high local thermal anomalies (above the TRPV1 threshold) may exist at the neurites or fibers' scale for these lower velocities. In the most extreme scenario, and for the fastest cracks, Equation (7) predicts temperature burst up to Δ*T*′ ~ ϕ*G*/(π*Cl*) ~ 100 − 1,000°C at the microscopic scale. Of course such temperatures may seem excessive in a biological context, but they were actually suspected in the rupture of various other materials (e.g., Fuller et al., 1975; Pallares et al., 2012; Toussaint et al., 2016; Vincent-Dospital et al., 2020b). These high temperatures could themselves cause a rapid deterioration of the surrounding skin cells (e.g., Xu and Lu, 2011), but they would only occur briefly and locally around an already failing tissue so that such potential secondary thermal damage may not be of key significance.

What remains certain is that milder temperature anomalies, but still strong enough to trigger the TRP nociceptors, can be directly measured.

### 4.3. *In vivo* vs. *Ex vivo* Skin

Although porcine skin is only a model of human skin, we have discussed, in section 1.2, how the orders of magnitude we report should be valid to the human biology, in particular as pig and human skin have relatively close mechanical strength and thermal properties (Giering et al., 1996; Debeer et al., 2013; Ranamukhaarachchi et al., 2016).

An additional complexity to consider, in order to assess the relevance of our results to live subjects, is that live skin is continuously blood-irrigated, which is a source for heat transport (advection) not at play in our experiments. In the skin capillaries, which are likely present locally around any rupture, an order of magnitude for the blood velocity at rest is *V*_*B*_ ~ 0.5 mm s^−1^ (e.g., Stücker et al., 1996). Even considering a perfect and instantaneous heat transfer between the structural tissue and such a blood network (a hardly realistic hypothesis), this would imply a smear of the dissipated heat over, at most, a distance τ*V*_*B*_ ~ 10μm, for the time interval τ = 20ms accessible to our camera. This non-conservative length scale is in the same range and actually slightly smaller than the skin depth of the simultaneous heat conduction in our samples $\sqrt{\lambda \tau /\left(\rho c\right)}\sim$40μm. Thus, we point out that our experimental lack of blood circulation shall not significantly undermine our core observations. For similar reasons, it is also unlikely that a hormonal regulation of the temperature, also bound to occur in an *in vivo* skin, can efficiently mitigate the transient heat signal we describe, as hormonal transport (either in the blood or by diffusion in the tissue—e.g., Berry et al., 2015) should be slow compared to the generation of a skin fracture.

Note however that, to some extent, further studies may target *in vivo* human samples (rather than *ex vivo* porcine ones), as skin rupture is a common medical practice and as infrared measurements are fully non-invasive.

### 4.4. On the Magnitude of the Heat Dissipation and Various Damage Types

In this manuscript, we gave a broad estimation, with two different methods, of the percentage of mechanical work that is effectively transformed into heat during the propagation of cutaneous cracks (ϕ~3–50%, with a mean representative value above 10%—see Table 1 and Figure 6). This portion being non negligible, it could make thermal monitoring a “natural” way for the detection of damages, and, as a very qualitative statement, it would not be surprising that evolution exploited the detection of the dissipated heat for the preservation of life. Note that, consistently, our estimation of ϕ is inline with what we elsewhere reported for the tear of another fibrous tissue of biological origin, that is, paper, where ϕ was measured between 10 and 40% (Toussaint et al., 2016).

The here measured thermal anomalies are however bigger than those that we recently theorised, when the discussed pain pathway was first proposed (Vincent-Dospital and Toussaint, 2021). In this former theoretical study, it was suggested that these anomalies should be on the edge of the TRPs sensitivity and thus relevant to hyperalgesia only rather than to direct algesia as well. By contrast, we have here shown that direct thermo-mechanical algesia is also likely at play for fast cracks. One of the differences between the previous theoretical work and the present one is that damages at the full skin scale have here been studied, while the rupture of a unique collagen fiber was priorly considered. This being stated, the main difference lies in the value of the considered energy release rate, that was here computed to be in the 80−210 kJ m^−2^ range for the tearing of skin, but reported to be more than one order of magnitude smaller for the cutting of skin (Pereira et al., 1997). Such discrepancy likely derives from the different role of inter fibre friction when tearing or cutting skin. Such inner friction was proposed to account for most of the cutaneous strength in tearing (Yang et al., 2015), but is likely negligible in cutting. Note that the tearing of porcine dermis, with different loading modes than the one that we here studied, can also dissipate less energy than our inverted mean energy release rate (Rodriguez et al., 2021). Similar studies to the present one should then be performed for other types of damages, and in particular for cuts or punctures which are both common injuries and common medical procedures. Thermo-mechanical pain may there be of different importance.

### 4.5. On Other Pain Mechanisms

It is important to state that the pain pathway that we here propose is not to comprehensively account for any sense of pain. For instance, the pressure pain threshold in human subjects was measured (e.g., Jensen et al., 1986) to be around 0.1 to 1 MPa, that is, at stress levels far less than what is needed to initiate an actual skin rupture and hence strong thermal anomalies [i.e., ${\bar{\sigma }}_{\text{f}}$ in the order of 10MPa, as here or elsewhere (Ní Annaidh et al., 2012) reported]. It is actually comforting that pain shall occur before an actual rupture, but shall also increase, maybe through different mechanisms, when rupture is indeed reached.

Other nociceptors exist at the membrane of neurons, for instance, the Piezo channels (Murthy et al., 2018), which opening is believed to be related to the stretch of cells' membranes. Such channel opening with stretch has, interestingly, also been proposed as another explanation for the involvement of TRPs in mechanical sensing (Liu and Montell, 2015), without the consideration of any thermal anomaly. In practice, both effects could coexist, with the thermal sensitivity of TRPs that could be improved by their abnormal stretch, hence leading to a polymodal detection.

Finally, in this work, we have only considered the triggering of heat sensitive TRPs as the mechanism for mechanical pain. One may additionally wonder about a similar involvement of the cold sensitive TRPs, such as TRPA1 and TRPM8, as an effect of these proteins on mechanical algesia has also been evidenced (e.g., Brierley et al., 2011; De Caro et al., 2018). Some cross-talk has been shown to take place between TRPs, for instance between TRPV1 and TRPA1 (Spahn et al., 2014), and a hypothesis could be that the abnormal activation of the former by fracture-induced heat may alter the responsiveness of the latter. In our experiments, we have not registered any local decrease of skin temperature around the tearing fractures and only measured a temperature rise, but such cold anomalies—which could directly trigger cold sensitive TRPs—have actually been reported in the rupture of other materials (Fuller et al., 1975; Rittel, 1998). Additionally, in the cutaneous tissue, the vasoconstriction under mechanical stress could also be prone to induce some local cooling (Rubinstein and Sessler, 1990).

Overall, mechanical algesia is bound to be a very convoluted phenomenon, involving many types of nociceptors and of biological processes (e.g., Hill and Bautista, 2020). The present study aimed to introduce and shed some—infrared—light on a new, thermo-mechanical, pain pathway.

## Data Availability Statement

The original contributions presented in the study are included in the article/Supplementary Material, further inquiries can be directed to the corresponding author/s.

## Author Contributions

TV-D proposed the pain theory, performed the experimental work, and wrote the first version of the manuscript. RT and KM advised on rupture mechanics and energy dissipation and on the experimental set-up. All authors contributed and agreed on the final version.

## Funding

The authors acknowledge the support of the Universities of Oslo and Strasbourg, of PoreLab, of the Njord centre and of the IRP France-Norway D-FFRACT. This work was also partly supported by the Research Council of Norway through its Centre of Excellence funding scheme, project number 262644.

## Conflict of Interest

The authors declare that the research was conducted in the absence of any commercial or financial relationships that could be construed as a potential conflict of interest.

## Publisher's Note

All claims expressed in this article are solely those of the authors and do not necessarily represent those of their affiliated organizations, or those of the publisher, the editors and the reviewers. Any product that may be evaluated in this article, or claim that may be made by its manufacturer, is not guaranteed or endorsed by the publisher.

## Figures and Tables

**Figure A1:**
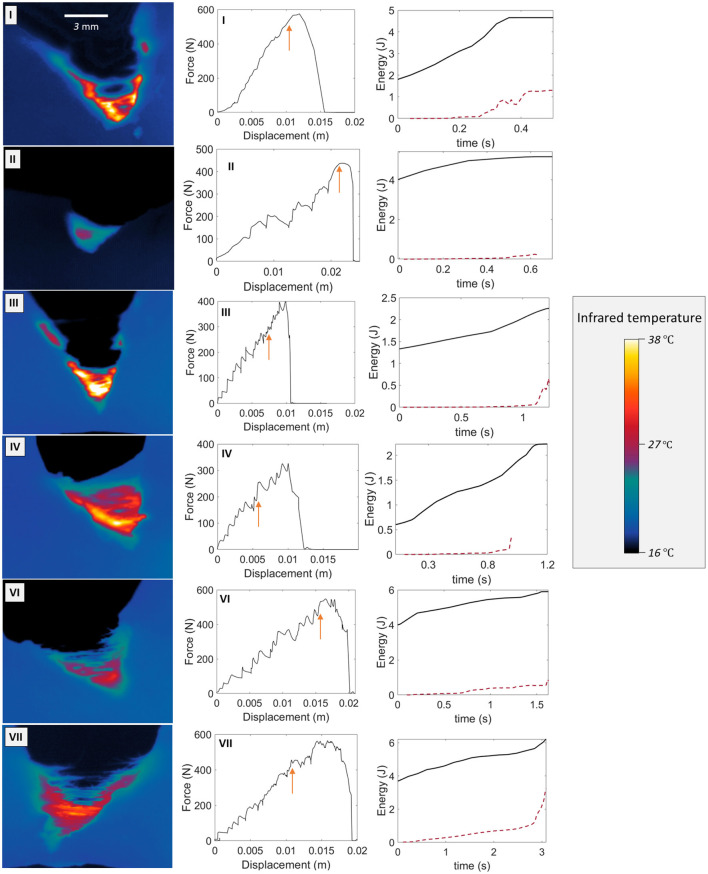
Temperature map examples (first column), force vs. displacement curves (second column), and time evolution of *W* and *U* (plain and dashed plot, respectively, third column) from the onset of the rupture (arbitrarily at *t* = 0 s) for skin specimens I, II, III, IV, VI, and VII. The arrows on the *F* vs. Δ*l* plots indicate the onsets of rupture.

**Figure A2:**
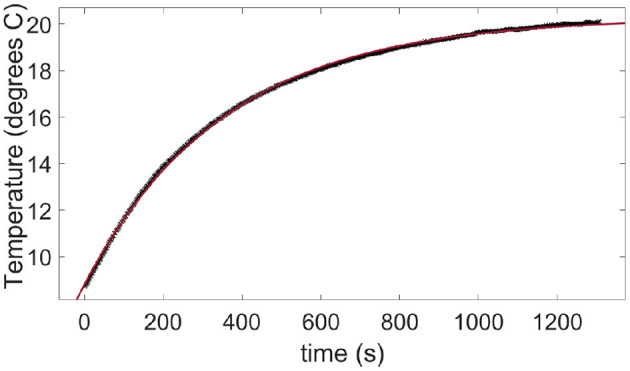
Warming up in air at room temperature of a skin sample taken out from a refrigerator. The data points (crosses) are measured with the infrared camera. The plain line is a fit of Equation (B1) with a time constant τ_a_ = 356 s.

## Appendices

### Other Skin Specimens

Figure A1 shows the experimental measurements for the skin specimens not fully presented in the manuscript (i.e., all specimens except sample V). A temperature map at an arbitrary time, the force vs. displacement plot and the time evolution of *W* and *U* after the onset of rupture are there represented.

### Thermal Exchange With the Ambient Air

Figure A2 shows the slow warming up of a skin sample taken out of a refrigerator and placed, in the ambient air, in front of the infrared camera. The following function is fitted to the temperature data points:

(10)
$$\begin{array}{l} T\left(t\right)=T_{\text{i}}+\text{Δ}T_{\text{max}}\left(1-\exp\left(-\frac{t}{\tau_{\text{a}}}\right)\right), \end{array}$$

where *T*_i_ is the initial sample temperature, Δ*T*_max_ is the final rise in temperature of the warning sample and τ_a_~6 min is the typical time constant for the air-skin thermal exchange. This time constant being about two orders of magnitude higher than the typical duration of our fracture tests (from 1 to 10s), the air-skin thermal exchange has been neglected in this manuscript.
